# Opportunities and risks of large language models in digital interventions for substance use disorders

**DOI:** 10.1097/YCO.0000000000001088

**Published:** 2026-04-29

**Authors:** Marissa de Vries, Michael P. Schaub

**Affiliations:** a Research Department Jellinek, Arkin, Amsterdam Netherlands; b Swiss Research Institute for Public Health and Addiction, associated to the University of Zurich, Zurich, Switzerland

**Keywords:** artificial intelligence, digital interventions, large language models, substance use disorder

## Abstract

**Purpose of review:**

Large language models (LLMs) are increasingly integrated into digital mental health tools, yet their role in substance use disorder (SUD) interventions remains poorly understood. This review synthesizes emerging evidence on the opportunities and risks of applying LLMs across the digital SUD care continuum.

**Recent findings:**

Studies report promising applications in early detection, personalized support, continuous monitoring, and relapse prevention. LLMs demonstrate capacity to extract substance-use signals from natural language, generate supportive and motivational responses, and interpret narrative data for patient-reported outcomes. However, risks are substantial. LLMs can produce inaccurate or hallucinated content, may reinforce stigma or demographic bias, and can generate misleading or potentially unsafe advice. Privacy concerns are amplified in SUD contexts, where sensitive data are often managed outside regulated healthcare systems. Existing regulatory frameworks such as the EU AI Act or U.S. device regulations, do not yet provide clear governance for anonymous, AI-supported SUD interventions.

**Summary:**

LLMs have potential to expand scalable, low-threshold support for SUDs, but their safe deployment requires validation, bias mitigation, transparent data governance, and robust human oversight. Evidence remains preliminary, and clinical integration should proceed cautiously.

## INTRODUCTION

Substance use disorders (SUDs) represent a major global health burden [1–3], with many individuals experiencing limited access to care due to stigma, costs, workforce shortages and long waiting times [4^▪^,^5^,6^▪^]. In response to this treatment gap, digital interventions have emerged as scalable solutions that can supplement traditional treatments [7,8^▪^]. Such digital interventions include mobile health (mHealth) applications, telehealth platforms, and web-based programs designed to support behavior change, monitor symptoms and enhance treatment engagement [7].

More recently, innovations in artificial intelligence (AI) have introduced new possibilities for digital mental health and addiction support. Within this rapidly evolving landscape, large language models (LLMs) – AI systems trained on vast amounts of text that can generate human-like language and engage in conversational dialogue – have gained growing attention for their potential roles in mental health and addiction care [9,10,11^▪^]. Emerging work in SUD-related contexts has explored LLM applications for early detection and screening [12,13^▪^,^14^], conversational and therapeutic support [9,15,16^▪^] and monitoring or relapse prevention [13^▪^,^17^,^18^]. To date, we are not aware of studies that evaluate LLMs as dedicated harm-reduction tools within SUD care. Nonetheless, emerging evidence shows that LLMs are capable of embedding harm-reduction as well as prevention principles into conversational interactions for example, by encouraging safer-use practices, offering guidance related to overdose prevention, and reducing stigmatizing language [17,19^▪^,20^▪^,^21^]. Together, this findings suggests that LLMs may hold promise across multiple points in the SUD treatment continuum.

At the same time, important risks must be acknowledged. Current LLMs are prone to inconsistencies and “hallucinations”, referring to the generation of information that appears plausible but is factually incorrect or unsupported by evidence. LLMs have also been shown to produce inaccurate, overly generalized, or biased responses that may disproportionately affect individuals with complex or marginalized health and social needs [22,23]. The dissemination of misleading information may contribute to incorrect assessments or unverified medical advice, posing threats to patient safety [9,23,24]. Beyond content accuracy, concerns also relate to data privacy and the broader ethical implications of deploying these systems in addiction care [16^▪^,^24^]. Taken together, these developments underscore the need to critically examine both the opportunities and risks of applying LLMs within digital interventions for SUDs, which is the focus of this review. 

**Box 1:**
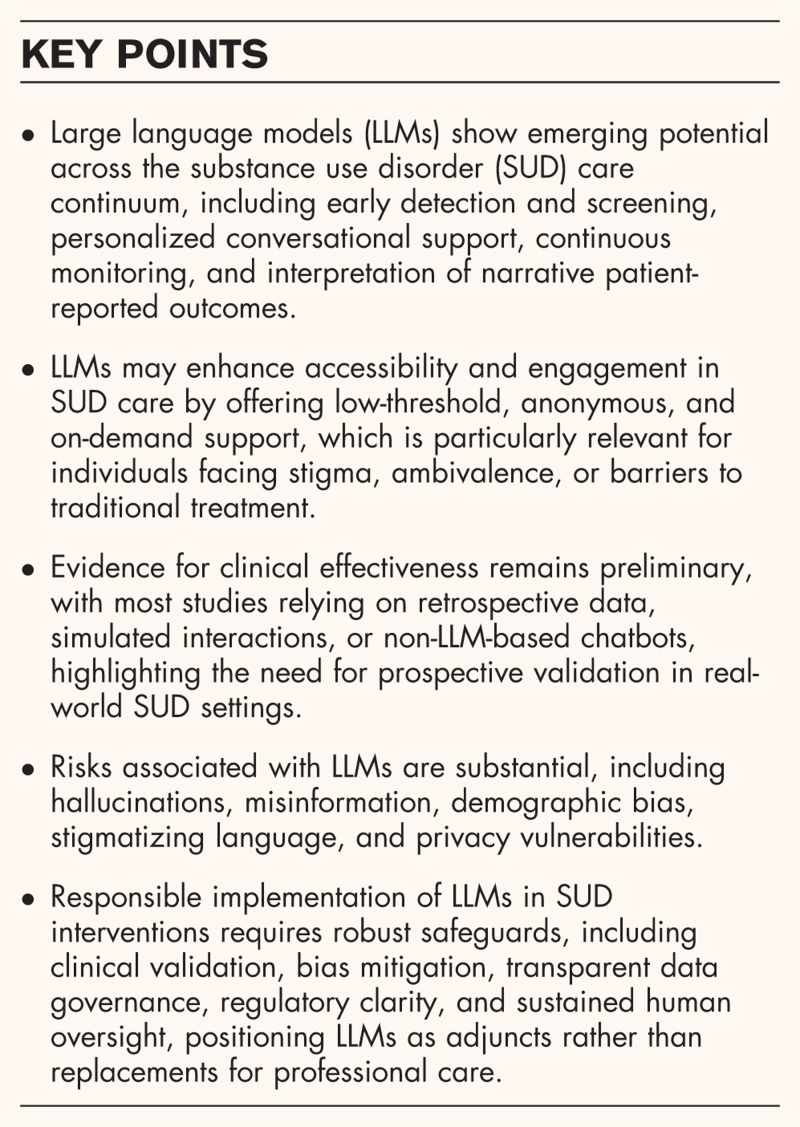
no caption available

## OPPORTUNITIES OF LARGE LANGUAGE MODELS IN SUBSTANCE USE DISORDER INTERVENTIONS

### Early detection, screening, and risk stratification

Early identification of problematic substance use is a persistent challenge in clinical practice, with many individuals remaining undiagnosed or entering treatment only at later, more severe stages [1–3]. LLMs offer new possibilities for enhancing early detection by analyzing natural language data from clinical encounters, screening tools, or digital platforms. For example, Giorgi *et al.* [12] found that generative AI models can extract substance-use–related information from real-world questions, revealing their capacity to recognize patterns of alcohol and drug use within user-generated text. Similarly, Mahbub *et al.* [14] showed that an LLM could classify SUD severity directly from clinical notes, outperforming traditional keyword-based or structured coding approaches, and demonstrating the value of free-text analysis for early identification.

Although these developments are promising, it is important to emphasize that most early-detection applications remain exploratory and lack prospective clinical validation. Current evidence is largely based on retrospective datasets or simulated queries, and few studies have assessed whether LLM-supported screening improves real-world outcomes. Nevertheless, the available literature suggests that LLMs have substantial potential to augment early detection, offering flexible, scalable approaches to identifying risk in individuals who may otherwise remain outside the formal treatment system.

### Personalized support and therapeutic dialogue

A central challenge in SUD treatment is sustaining engagement and providing consistent psychosocial support, particularly given widespread barriers to in-person counselling such as stigma, cost, and limited availability of trained professionals [4^▪^,^5^,6^▪^]. LLMs offer potential value in this domain by enabling conversational interactions that resemble elements of therapeutic communication. Systematic reviews of LLM-based mental health applications indicate that these models can generate empathic-like, supportive responses, deliver psychoeducation, and engage users in motivational or reflective dialogue [9,10]. Because LLMs are able to adapt tone, complexity, and content to the user's linguistic style, they may help create a personalized and non-judgmental interaction environment which are important ingredients for enhancing engagement among individuals hesitant to seek formal care [9,16^▪^].

In SUD-specific contexts, chatbot-assisted interventions have shown promising early results. A systematic review by Lee *et al.* [15] demonstrates that conversational agents – primarily delivered as stand-alone or minimally supported digital interventions – can reduce substance use, increase readiness to change, and support harm-reduction behaviors, although most systems reviewed were rule-based rather than LLM-driven. Newer LLM-enabled systems extend these capabilities by offering more naturalistic and flexible dialogues, which may more closely approximate components of motivational interviewing or cognitive-behavioral strategies [25].

The potential benefits of LLM-based conversational support may be particularly relevant for individuals who experience shame, fear of judgment, or ambivalence toward treatment. Factors known to reduce help-seeking in SUD populations [3,16^▪^]. By offering anonymous, stigma-free, and on-demand interactions, LLMs may facilitate early engagement and increase user comfort [9,11^▪^]. Although formal evidence within blended SUD treatment is still limited, CAs have demonstrated the capacity to provide between-session reinforcement and motivation in digital mental-health and substance-use interventions [9,10,15,16^▪^,^25^], suggesting similar potential in blended care models. However, these systems should not be viewed as replacements for trained clinicians. Concerns related to misinformation, emotional over-reliance, and the absence of professional oversight underscore the need for cautious integration and rigorous evaluation before LLM-assisted dialogue can be considered a reliable component of SUD care [12,24].

### Language-based monitoring, relapse prevention, and patient-reported outcomes

The fluctuating nature of cravings, stress, and motivation makes continuous monitoring an important component in SUD care [4^▪^,^5^]. LLMs offer new possibilities for supporting relapse prevention by analyzing users’ natural language inputs such as daily check-ins, craving logs, or reflective journaling to detect emerging changes in mood, stress, or substance-use risk. A recent scoping review identified monitoring and self-management as dominant use cases of AI-driven digital interventions, with LLMs increasingly being incorporated into these functions [18].

SUD-focused evidence supports the potential value of language-derived markers in predicting disengagement and relapse-related outcomes. Curtis *et al.* [13^▪^] demonstrated that linguistic features from social media language predicted treatment dropout, suggesting that real-time language monitoring could identify individuals needing additional support.

LLMs may also advance the development and interpretation of patient-reported outcomes (PRO). Traditional PRO instruments often lack sensitivity to the contextual and moment-to-moment variations typical of SUD trajectories. Generative AI offers the possibility of “language-native” PROs that synthesize narrative input into clinically meaningful insights. Boyer *et al.* [17] propose that such tools could yield more personalized information than fixed-item questionnaires, potentially supporting more dynamic, responsive treatment planning.

Overall, LLM-driven monitoring and PRO-based tools show significant promise for personalized relapse-prevention strategies. However, current evidence is preliminary, and prospective clinical studies are needed to determine whether these systems improve real-world outcomes such as relapse rates, engagement, or treatment adherence.

## RISKS AND UNCERTAINTIES OF LARGE LANGUAGE MODELS IN SUBSTANCE USE DISORDER INTERVENTIONS

### Reliability and clinical safety

Despite their increasingly sophisticated conversational abilities, LLMs remain vulnerable to important reliability and clinical safety limitations. A central concern is their tendency to generate responses that appear coherent and authoritative while containing factual errors, omissions, or unfounded assertions. In the LLM literature, such incorrect but persuasive outputs are commonly referred to as “hallucinations”. Hallucinations are model-generated statements that do not accurately reflect underlying evidence or established knowledge. Reviews of LLMs in mental health applications consistently note that these hallucinations, together with more general issues of inaccuracy and limited verifiability, continue to constrain the safe use of such systems in clinical or clinically adjacent contexts [9,10,15].

Evidence specific to substance use contexts reflects similar risks. Giorgi *et al.* [12] found that LLM-generated answers to real-world drug-use and harm-reduction queries were at times incomplete, misleading, or potentially unsafe. Spallek *et al.* [11^▪^] likewise reported variable accuracy and occasional misinformation in ChatGPT's content related to mental health and substance use.

A further challenge is the models’ tendency to present their outputs with a high degree of confidence, regardless of factual correctness. This “overconfidence effect” may lead users to over-estimate the reliability of model responses, particularly in situations where individuals seek rapid, anonymous guidance during distress, uncertainty, or withdrawal. As Blease and Torous [16^▪^] note, conversational fluency can create an impression of competence that may not reflect the underlying reliability of the model.

Overall, current findings indicate that reliability and clinical safety remain active areas of concern, and that further empirical validation is required to clarify the conditions under which LLMs can be integrated safely and responsibly into SUD-related settings.

### Fairness and stigma

Fairness and stigma are central concerns when deploying LLMs in digital interventions for SUDs. People with SUDs already face high levels of structural stigma, inequity in access to care, and negative stereotyping within healthcare systems [5]. If LLMs reproduce or amplify these patterns, even subtly, they may undermine engagement, reinforce barriers to treatment, or misinform users seeking support.

A growing body of research shows that LLMs do not consistently use person-first or non-stigmatizing language in SUD contexts. Wang *et al.* [22], who evaluated multiple LLMs across a range of alcohol- and substance-use prompts, found frequent use of stigmatizing labels such as “addict” and “alcoholic,” particularly when such terms appeared in user input. Although prompt-engineering techniques reduced the frequency of stigmatizing language, the authors emphasize that the underlying behavior remains inconsistent and highly sensitive to wording. Hackl [26] reports a similar pattern: while LLMs often adopt person-first language for conditions such as depression or anxiety, they are far less reliable when discussing addiction, sometimes introducing non-inclusive terminology spontaneously. Together, these findings suggest that LLMs can produce destigmatizing language but do not do so reliably without explicit constraints.

The consequences of such inconsistency are not merely linguistic. Mittal *et al.* [27] demonstrate that exposure to AI-generated content can directly influence users’ attitudes toward individuals with opioid use disorder. In their experiments, stigmatizing text increased punitive attitudes and reduced support for treatment, whereas destigmatizing text led to more compassionate responses. Because LLMs can inadvertently generate biased or stigmatizing phrasing, this work implies that AI-driven SUD tools have the potential to shape public perceptions in ways that may either support or hinder (digital) treatment engagement.

Fairness concerns further extend to differences across demographic groups. Ong *et al.* [23] highlight how AI systems can reproduce socioeconomic and structural inequities embedded in their training data. Empirical work in mental health demonstrates that LLM-generated assessments can vary systematically by gender or other demographic markers even when clinical information is held constant [28], while systematic reviews by Guo *et al.* [9] and Bucher *et al.* [10] similarly report demographic sensitivity in model outputs. In SUD contexts, where outcomes are strongly shaped by social determinants of health, many such biases influence how different groups are assessed or supported, potentially affecting the consistency of SUD care.

While emerging benchmarks and evaluation frameworks exist for assessing stigma and bias [29], these tools are not yet widely implemented in real-world SUD interventions. Fairness and stigma therefore require explicit design choices, continuous monitoring, and involvement of people with lived experience.

### Privacy, data protection and regulation

Privacy and data protection are particularly important in digital interventions for SUDs, as individuals with substance use problems face elevated risks of stigma, discrimination, and social harm [5]. Disclosure of substance use information can affect employment, insurance, parental rights, and legal outcomes. Many individuals therefore seek digital and AI-supported tools because they offer anonymity and low-threshold access outside formal treatment pathways [7,8^▪^].

LLM-based systems raise specific privacy concerns. Most models rely on cloud-based infrastructures, meaning user inputs are transmitted to external servers. Users often lack clarity about whether conversational data are stored, reused, or shared with third parties [16^▪^,^23^]. Even when anonymized, SUD-related information such as substance-use patterns or relapse experiences, can be highly sensitive and potentially identifiable, particularly in small or marginalized populations.

Regulatory oversight remains limited because many LLM-based SUD tools operate outside formal healthcare systems. Consumer-facing chatbots and self-guided applications are often not classified as medical devices and therefore fall outside established clinical governance and health-specific data-protection frameworks [30,31]. Although initiatives such as the EU Artificial Intelligence Act and U.S. regulatory pathways represent important progress, they were primarily developed for traditional medical technologies or general-purpose AI and do not fully address the risks posed by anonymous, conversational tools used in SUD contexts.

As a result, several specific regulatory gaps remain unaddressed. These include: classification, as it remains unclear whether LLM-based SUD chatbots should be regulated as medical devices or consumer tools; privacy and secondary data use, given the lack of clear minimum standards for data storage, reuse, and transparency in anonymous AI-supported interventions; responsibility and liability, particularly when AI-generated advice is inaccurate or causes harm in non-clinical settings; post-deployment monitoring, including requirements for ongoing assessment of safety, bias, and performance; and safeguards for vulnerable and stigmatized populations, who face heightened risks when engaging with digital SUD tools.

Together, these gaps highlight the need for more SUD-specific regulatory guidance that clarifies expectations around data governance, accountability, and ongoing oversight, while recognizing the role of anonymous digital tools in substance use support.

## CONCLUSION

LLMs offer meaningful opportunities to strengthen digital interventions for SUDs, particularly in early detection, personalized support, continuous monitoring, and relapse prevention [9,12,13^▪^,^14^,^15^,16^▪^,^17^,^18^,^22^]. Current evidence suggests that LLMs can enhance accessibility, provide empathetic and low-threshold engagement [9,10,15,16^▪^], and help identify emerging risk patterns from natural language that might otherwise remain unnoticed [12,13^▪^,^14^,^17^,^18^]. Yet these benefits remain largely theoretical or demonstrated only in controlled or retrospective settings, and real-world clinical effectiveness has not yet been firmly established [9,10,15].

At the same time, the risks presented by LLMs are substantial. Reliability concerns including hallucinations, inaccuracies, and overconfident presentation of incorrect information, pose direct threats to clinical safety [9,10,11^▪^,^12^,16^▪^,^22^]. Bias and stigmatizing language remain pervasive, with multiple studies showing that LLM outputs can reproduce harmful terminology or demographic variability even when clinical information is held constant [19^▪^,20^▪^,^22^,^26^,^27^]. Privacy and data-governance challenges are heightened in SUD contexts, where disclosure of sensitive information carries disproportionate consequences and many tools operate outside formal healthcare systems with limited oversight [7,8^▪^,16^▪^,^24^]. Existing regulatory frameworks – such as the EU AI Act and U.S. HIPAA and FDA pathways – do not yet provide specific governance for anonymous, AI-driven SUD support [29–31].

The way forward requires moving beyond a binary of opportunity versus risk. Progress depends on establishing SUD-specific safety benchmarks [22,27], improving transparency around data practices [16^▪^,^24^], mitigating hallucinations and bias through technical safeguards [9,10,22], and embedding LLM tools within clinical ecosystems that ensure human oversight [16^▪^,^21^].

Involving people with lived or living experience of substance use should be viewed as a key safeguard in the development and deployment of LLM-based interventions. Their perspectives are essential for identifying stigmatizing language, unsafe assumptions, and design blind spots that may be overlooked by developers or clinicians. Meaningful involvement can occur across stages such as co-design, risk assessment, validation, and real-world implementation, helping to reduce inequities, build trust, and ensure alignment with the needs of people seeking support for substance use.

Overall, LLMs have the potential to complement, but not replace, traditional SUD care. Their responsible implementation will require continued evaluation, interdisciplinary governance, and ongoing monitoring to ensure that digital innovation enhances, rather than compromises, the welfare of individuals seeking support for substance use.

## Acknowledgements

*None.*

### Financial support and sponsorship

*None.*

### Conflicts of interest

*There are no conflicts of interest.*

## REFERENCES AND RECOMMENDED READING

Papers of particular interest, published within the annual period of review, have been highlighted as:

▪ of special interest
▪▪ of outstanding interest
